# Health and economic impact associated with rheumatoid arthritis discharges: a cost analysis of a two-year cohort in Mexico

**DOI:** 10.1186/s12913-023-10298-w

**Published:** 2023-11-29

**Authors:** Carlos Fernando Mendoza-Gutierrez, Diana Montiel-Ojeda, Delfino Vargas-Chanes, Nelly Cisneros-González, José Esteban Fernández-Gárate, Blanca Godina-Ortiz, Patricia Clark

**Affiliations:** 1 Clinical Epidemiology Research Unit, Children’s Hospital of Mexico Federico Gomez-Faculty of Medicine, National Autonomous University of Mexico UNAM, Mexico City, 06720 Mexico; 2 Programa Universitario de Estudios del Desarrollo, National Autonomous University of Mexico UNAM, Mexico City, 04510 Mexico; 3 Epidemiological Surveillance Coordination, Instituto Mexicano del Seguro Social, Mexico City, 03100 Mexico; 4 Palagod Health Supply SA de CV, Mexico City, Mexico

**Keywords:** Cost analysis, Rheumatoid arthritis, Healthcare systems, Public health, Mexico

## Abstract

**Background:**

Rheumatoid arthritis is a highly prevalent disease. Patients undergo various medical and pharmacological treatments, which have an economic impact on hospitals. The aim of this study was to estimate the direct economic costs of Mexican Social Security Institute (IMSS) resources used to provide healthcare to adult patients with rheumatoid arthritis in 2016–2017.

**Methods:**

Data of patients aged > 18 years with Rheumatoid Arthritis (RA) were obtained from databases and public information sources to estimate the use of IMSS resources for the target population. Total costs were estimated by means of the macro-costing method, employing the diagnosis-related group (DRG). Each DRG of the IMSS was constructed with one of the diagnoses and the respective combination of clinical characteristics included in the ICD-9. This study was conducted from the national perspective of IMSS, the largest healthcare service administrator in the country. As such, it can be considered representative of the broader healthcare landscape in Mexico.

**Results:**

The total cost per year of furnishing inpatient care to RA patients was found to be $170,099,794 MXN ($9,096,245.67 USD) for 2016 and $167,039,481 MXN ($8,932,592.57 USD) for 2017, implying an enormous economic impact on the government budget for Mexican public health services.

**Conclusions:**

Our results demonstrate that the direct costs of musculoskeletal and cardiovascular surgery represented the highest costs of RA in-hospital care at IMSS (the largest health institution in Mexico) in 2016 and 2017. Further studies are needed that include the cost of drugs and other indirect costs in addition to our results to get the most accurate approximation of the cost of living with RA.

**Supplementary Information:**

The online version contains supplementary material available at 10.1186/s12913-023-10298-w.

## Background

Rheumatoid arthritis (RA) is a prevalent disease worldwide, affecting 1.2 people per 100 individuals [1]. Although, the average prevalence in Latin America is ~ 0.5 cases per 100 inhabitants [2], it is higher in Mexico, being ~ 1.6 cases per 100 inhabitants [3]. RA is usually diagnosed at the age of 47.8 ± 15.5 years of age and this patients can live an estimated of 15 to 30 years with the disease [4]. According to a report in 2017, disorders of the musculoskeletal system (which includes the RA) worldwide generated approximately 138.7 million of disability-adjusted life years (DALYs) each year (IC 95%, 82.6-101.9 million) [5]. In Mexico, DALYs increased by 41% from 1990 to 2016 due to the increase in these musculoskeletal disorders [6].

Numerous pharmacological treatments are available for patients with RA [5]. The standard initial treatment is based on conventional synthetic disease-modifying antirheumatic drugs (csDMARDs). In patients with moderate RA and an inadequate response to csDMARDs after 12 weeks of treatment, the use biologic DMARDs (boDMARDs) is recommended. These drugs are also suggested for patients with RA who present risk factors associated with a bad prognosis at the moment of diagnosis [7]. The boDMARDs consist of tumor necrosis factor inhibitors (TNFi), interleukin 6 receptor inhibitors (IL-6i), and janus kinase inhibitors (JAFi).

The average time of treatment of a patient with RA is five years [8]. According to the guidelines for clinical practice of the American College of Rheumatology (2021), patients with RA take an average of three DMARDs as of the beginning of treatment [9], which translates into a great economic impact. It has been reported that the annual cost of treating RA is estimated at $36,053 per patient, representing a burdensome economic impact for health systems [4, 10]. Additionally, the chronicity of RA tends to engender therapeutic failures or inadequate responses to treatment (IRT). IRT in RA is defined as the continuation of an accelerated rate of pathogenesis (determined by the DAS28 index and a positive PCR test, as outlined in *Musculoskeletal Guidelines: Rheumatoid Arthritis*). The resulting problems of mobility require a change of therapy, physical rehabilitation, surgery, or some other type of intervention [11], implying an even greater use of hospital resources [12].

In Mexico, around 30% of patients with RA have an IRT, which leads to a considerable number of hospitalized patients and consequently to a high average cost of treatment for the disease [13, 14]. Although there are reports on the economic impact of drug treatment of patients with RA [15], little is known about the economic costs of hospital resources dedicated to these patients.

For decision makers, it is important to ascertain the costs of drug treatment and hospital services associated with any disease. For chronic diseases such as RA, the capacity to appraise the specific cost of medical attention is essential for long-term planning. Therefore, the objective of this study was to estimate the direct economic costs incurred by the IMSS in providing in-patient interventions and care for adult patients with rheumatoid arthritis during the years 2016–2017. The IMSS is the Mexican Institute of Social Security (Instituto Mexicano del Seguro Social), the largest public healthcare service in the country.

## Methods

### Setting

We conducted a cost-analysis study to estimate the direct economic costs of the resources used to provide healthcare for patients with RA. To this, the present study was conducted on adults (over 18 years old) diagnosed with RA based on the criteria of the American College of Rheumatology [16], contemplating morning stiffness, arthritis in the hands and/or in at least three joint areas, symmetrical arthritis, Heberden’s nodes, serum rheumatoid factor, and/or radiographic changes. The population of participants consisted of RA patients who received medical attention in one of the Highly Specialized Medical Units of the IMSS from 2016 to 2017. The IMSS is the largest and most representative healthcare system in Mexico, giving medical attention to 6 of 10 citizens [17]. Therefore, the information gathered in this institution can be generalized to most of the country’s population. This study primarily examined direct costs, adopting the institutional payer’s perspective.

### Databases

Data was obtained from the following databases and public sources of information in order to estimate the use of IMSS resources for the target population.

- ICD-10: The International Classification of Diseases, 10th edition: Identification and classification of the different medical-surgical procedures.
- System of Analysis of Non-communicable Diseases (SANENT, according to the initials in Spanish) of the IMSS: Identification of the beneficiaries of the IMSS that required some hospital intervention related to RA from 2016 to 2017.
- System of Information on Comprehensive Healthcare and the hospital records of the Medical Statistics System (DataMart) of the IMSS: Determination of the demographic characteristics and clinics of the patients with RA selected to participate in the study.
- Database of the Classification of Procedures of the ICD-9-CM (International Classification of Disease, 9th edition, Clinical Modification): Determination of the medical interventions and/or procedures carried out for each disorder of the participants.
- Diagnosis-related group (DRG): Estimation of the costs of the medical interventions performed.

### Procedures

All recorded cases of RA in adults over 18 years of age were identified in the SANENT® database, based on two categories (M05 and M06) of the ICD-10 [18]. The information on each participant consisted of the report number or identification number, the hospital unit, the administrative region, the diagnosis upon admission, and the specialty of the attending physician. Other information gathered (from the System of Information of Comprehensive Healthcare and the hospital records of DataMart of the IMSS) included sociodemographic data, the main diagnosis of each patient (in the event of more than one pathology, the disorder responsible for the greatest use of resources of the IMSS), the hospital services employed, the number of days of the hospital stay, and the dates of admission and discharge.

The medical interventions performed on each patient were identified with the database of the Classification of Procedures of the ICD-9-CM (published by the Secretary of Health of Mexico [19]), which has the following sixteen categories.

1. Operations on the nervous system.
2. Operations on the endocrine system.
3. Eye operations.
4. Ear operations.
5. Operations on the mouth, nose, or throat.
6. Operations on the respiratory apparatus.
7. Operations on the cardiovascular apparatus.
8. Operations on the hematic and/or lymphatic system.
9. Operations on the digestive apparatus.
10. Operations on the urinary apparatus.
11. Operations on the male genital organs.
12. Operations on the female genital organs.
13. Obstetric procedures.
14. Operations on the musculoskeletal apparatus.
15. Operations on the integumentary apparatus.
16. Physical therapy and rehabilitation.

### Statistical analysis

The population was divided into two groups in accordance with the year of their hospital discharge (2016 or 2017). The results were also analyzed in function of gender and total population. The distribution of the data was examined by utilizing the Kolmogorov-Smirnov test of normality with the frequentist approach. As a measure of the central tendency, the mean of the distribution was determined and expressed as an absolute value and as a percentage of the total participants. Regarding the clinical and sociodemographic characteristics, the mean difference between groups was evaluated with a two-sample Student’s *t*-test, assuming equal variance. The distribution of the variables was contrasted between genders.

1. Cost analysis.

We used the Diagnosis-Related Groups (DRGs) method to categorize and evaluate the costs associated with intrahospital treatment of rheumatoid arthritis. DRGs systematically grouped patients with similar diagnoses and treatment needs, providing a structured framework for cost estimation. This method facilitated the analysis of resource utilization and financial aspects within the context of intrahospital rheumatoid arthritis treatment [20].

The use of resources and the total costs were estimated by means of the macro-costing method, employing the DRG technique and taking as a reference the values reported by the hospital product (2017) of the IMSS. Each DRG of the IMSS was constructed with one of the diagnoses and the respective combination of clinical characteristics included in the ICD-9. Each one incorporates in a structured manner (as a factor) the type and quantity of resource necessary for specific functions [21].

The sequence of steps for examining the direct medical costs by DRG are presented hereafter.

1.1  Identification of the procedures applied to each patient.

A determination was made of the procedures and surgical interventions received by each patient during his or her hospital stay. In the database, the variable “main surgical intervention” recovers the classification code of procedures (ICD-9-CM) assigned to each patient diagnosed with RA and hospitalized.

1.2  Identification of the DRG associated with each patient.

Each ICD-9-CM code is related to at least one DRG. For each participant, the DRG defined at the time of hospital discharge served to cover the costs of the hospital stay. In the event the ICD-9-CM code was related to two or more DRGs, the one linked to less comorbidities and lower associated costs was chosen. In this way, the risk of overestimating costs was reduced.

1.3  Calculation of the cost of the DRG for each patient.

The relative weight of each procedure was determined in accordance with the DRG previously defined for each patient. The RW value was multiplied by the technical medical cost of reference specified by the IMSS, which for 2017 was established as $46,860.53 MXN, corresponding to DRG 868 (referring to the treatment of “other diagnoses of infectious and parasitic disease”). The product of the multiplication is considered the discharge cost for each patient, as shown in Eq. 1:

1 $$DCPi\ =\ (RW)\ast(TMC)$$

*DCPi: discharge cost of patient i*

*RW: relative weight*

*TMC: Technical medical cost (cost of one DRG).*

1.4  Cost updating.

Given that the information on costs was updated most recently in June of 2017, the costs were herein updated to January of 2023 in order to put the result in context. This task was accomplished by taking the inflation of the period into account, relying on the data reported by the National Institute of Statistics and Geography (Instituto Nacional de Estadística y Geografía, or INEGI). The product of the multiplication is considered the updated cost for each patient, as shown in Eq. 2:

2 $$UCPi\ =\ (ICPi)\ast(IPU)$$

*UCPi: updated cost of patient i*

*ICPi: Initial cost of patient i*.

*IPU: Inflation of the period to be updated.*

We converted the costs from Mexican pesos to US dollars using the official average exchange rate during the assessed period, which was (1 USD = 18.7 MXN).

## Results

In 2016 and 2017, a total of 3,623 and 3,427 RA patients (respectively) were hospitalized for some type of procedure at IMSS. For both years, the most prevalent age group was 50–69 years old. Among hospital admissions of RA patients 81% were women. Of all RA admissions recorded in the ED, 90% were referred to rheumatology and surgery. Less than 10% of the population were referred to another specialist on their first visit. The hospital discharge during 2016–2017 was due to clinical improvement in 90.3% of the cases. The deaths registered were less than 0.5% of the total hospitalized cases (Table 1).

**Table 1:** Demographic characteristics of the population selected, according to gender and year

	2016				2017			
	Total	Female	Male	p-value	Total	Female	Male	p-value
n (%)	3623 (100)	2944 (81.3)	679 (18.7)		3427 (100)	2779 (81.1)	648 (18.9)	
**Age**								
Under 30	163 (4.5)	123 (4.2)	40 (5.9)	0.8637	149 (4.4)	104 (3.7)	45 (6.9)	0.1103
30–39	335 (9.2)	278 (9.4)	57 (8.4)		331 (9.7)	290 (10.4)	41 (6.3)	
40–49	654 (18.1)	526 (17.9)	128 (18.9)		633 (18.5)	520 (18.7)	113 (17.4)	
50–59	898 (24.8)	753 (25.6)	145 (21.4)		786 (22.9)	659 (23.7)	127 (19.6)	
60–69	857 (23.7)	684 (23.2)	173 (25.5)		788 (23.0)	634 (22.8)	154 (23.8)	
70–79	523 (14.4)	422 (14.3)	101 (14.9)		561 (16.4)	426 (15.3)	135 (20.8)	
Over 79	193 (5.3)	158 (5.4)	35 (5.2)		179 (5.2)	146 (5.3)	33 (5.1)	
**Type of admission**								
Emergency	2,308 (63.7)	1,855 (63.0)	453 (66.7)	0.1856	2,131 (62.2)	1,703 (61.3)	428 (66.0)	0.0230*
Programmed	1,315 (36.3)	1,089 (37.0)	226 (33.3)		1,296 (37.8)	1076 (38.7)	220 (34.0)	
**Admission assignment**								
Rheumatology	1,240 (34.2)	1,015 (34.5)	225 (33.1)	0.2993	1,185 (34.6)	971 (35.0)	214 (33.0)	0.6455
Other	328 (9.1)	278 (9.4)	50 (7.4)		153 (4.5)	134 (4.8)	19 (2.9)	
Surgery	2,055 (56.7)	1,651 (56.1)	404 (59.5)		2,089 (61.0)	1,674 (60.2)	415 (64.0)	
**Motive of discharge**								
Not codified	145 (4)	134 (4.6)	11 (1.6)	0.1306	113 (3.3)	98 (3.5)	15 (2.3)	0.0825
Healed	6 (0.2)	5 (0.2)	1 (0.1)		2 (0.1)	1 (0.0)	1 (0.2)	
Withdraw	32 (0.9)	21 (0.7)	11 (1.6)		18 (0.5)	9 (0.3)	9 (1.4)	
Voluntary discharge	28 (0.8)	23 (0.8)	5 (0.7)		17 (0.5)	14 (0.5)	3 (0.5)	
Death	14 (0.4)	11 (0.4)	3 (0.4)		4 (0.1)	3 (0.1)	1 (0.2)	
Improvement	3290 (90.8)	2661 (90.4)	629 (92.6)		3,165 (92.4)	2,571 (92)	594 (91.7)	
Outpatient follow-up	108 (3)	89 (3)	19 (2.8)		108 (3.2)	83 (3.0)	25 (3.9)	

*Significant difference in the distribution of the variable using the method of means difference, with p < 0.05

Regarding the sixteen types of procedures for medical attention to RA patients (Table 2; Supplementary Fig. 1), the analysis revealed that three medical procedures were the most frequent, consuming 90% of the total resources. The lab and diagnostic tests represented the greatest expense, being responsible for 69.3% of total costs in both 2016 and 2017. In second place were the procedures (mostly surgical) on the musculoskeletal apparatus, constituting 19.1% and 18.7% of total costs in 2016 and 2017, respectively. The third costliest category was the diagnostics and miscellaneous, which was physical therapy and rehabilitation, comprising 2.6% and 3.2% of total costs in 2016 and 2017, respectively. When the results were analyzed by gender, the distribution of the distinct procedures was not significantly different between men and women in 2016 or in 2017 (p = 0.2830 and 0.2827, respectively), nor was there a significant difference in the total procedures between 2016 and 2017 (p = 0.9486).

**Table 2:** Type of procedure and the specialist responsible for the medical attention of the hospitalized patient

Type of procedure*	Hospital service	2016			2017		
		Female	Male	Total	Female	Male	Total
		n (%)	n (%)	n (%)	n (%)	n (%)	n (%)
Lab tests and other diagnostic tests	All	2,012 (68.3)	500 (73.6)	2,512 (69.3)	1,882 (67.7)	455 (70.2)	2,337 (68.2)
Musculoskeletal apparatus^+^	Rheumatology	577(19.5)	115 (16.5)	692 (19.1)	520 (18.7)	121 (18.7)	641 (18.7)
Physical therapy and rehabilitation^++^	Not specified	87 (3.0)	7(1)	94 (2.6)	99 (3.6)	9 (1.4)	108 (3.2)
Digestive apparatus	Gastroenterology	79 (2.7)	14 (2.1)	93 (2.6)	73 (2.6)	14 (2.2)	87 (2.5)
Integumentary apparatus	Dermatology	41 (1.4)	12 (1.8)	53 (1.5)	37 (1.3)	12 (1.9)	49 (1.4)
Eyes and annexes	Ophthalmology	37 (1.4)	9 (1.4)	46 (1.4)	38 (1.4)	9 (1.4)	47 (1.4)
Cardiovascular apparatus	Cardiology	41 (1.4)	7 (1.0)	48 (1.3)	34 (1.2)	9 (1.4)	43 (1.3)
Female genital apparatus	Gynecology	27 (0.9)	0 (0.0)	27 (0.7)	32 (1.2)	0 (0.0)	32 (0.9)
Nervous system	Neurology	21 (0.7)	7 (1)	28 (0.7)	17 (0.6)	6 (0.9)	23 (0.7)
Urinary apparatus	Urology	9 (0.3)	2 (0.3)	11 (0.3)	11 (0.4)	4 (0.6)	15 (0.4)
Mouth, nose, and throat	Otorhinolaryngology	20 (0.7)	3 (0.4)	23(0.6)	20 (0.7)	5 (0.8)	25 (0.8)
Hematic and lymphatic systems	Hematology	3 (0.1)	1 (0.1)	4(0.1)	6 (0.2)	0 (0.0)	6 (0.2)
Ears and annexes	Otorhinolaryngology	2(0.1)	2 (0.3)	4(0.1)	1 (0.1)	1 (0.2)	2 (0.1)
Endocrine system	Endocrinology	7 (0.2)	1(0.1)	8 (0.2)	4 (0.1)	0 (0.0)	4 (0.1)
Male genital apparatus	Urology	0 (0.0)	3 (0.4)	3 (0.1)	0 (0.0)	3 (0.5)	3 (0.1)
Obstetric procedures	Obstetrics	2 (0.1)	0.0 (0)	2 (0.1)	2 (0.1)	0.0	2 (0.1)

^*^All types of procedures, surgical and otherwise

^+^These procedures are mostly surgical, including repair of muscles, tendons, fasciae, and synovial membrane, as well as surgical reductions, joint dislocation, arthroscopy, etcetera

^++^ The IMSS category is “diagnostic tests and miscellaneous”, a misleading title

The category of inpatient procedures requiring the greatest expense for the IMSS was operations on the musculoskeletal apparatus, generating a cost of $132,053,692 MXN and $130,144,272 MXN during 2016 and 2017, respectively. The second costliest item of inpatient procedures was the category of surgical interventions on the cardiovascular system, even though such interventions are performed at a low annual frequency (1.3%). The economic impact was about 11.8 million MXN in 2016 and 11.5 million MXN in 2017. It turns out that most cardiovascular surgery had the purpose of attending to acute coronary syndrome, implying a high unit cost (Table 3).

**Table 3:** Costs of inpatient procedures associated with rheumatoid arthritis

System	2016			2017		
	Female	Male	Total	Female	Male	Total
Surgical procedures on the musculoskeletal apparatus	$113,512,090	$18,541,601	$132,053,692	$107,780,766	$22,363,504	$130,144,272
Physical therapy and rehabilitation^++^	$277,503	$22,328	$299,831	$315,779	$28,707	$344,486
Operation on the cardiovascular apparatus	$10,050,733	$1,830,560	$11,881,293	$8,790,406	$2,707,484	$11,497,891
Operation on the digestive apparatus	$7,219,852	$1,525,305	$8,745,158	$6,484,154	$1,736,194	$8,220,349
Operation on the integumentary apparatus	$4,214,137	$1,365,283	$5,579,421	$3,773,143	$1,426,888	$5,200,030
Operation on the mouth, nose, or throat (respiratory)	$2,102,679	$693,306	$2,795,986	$1,924,219	$668,015	$2,592,234
Eye operation and related procedures	$1,648,511	$384,119	$2,032,630	$1,688,003	$384,118	$2,072,122
Operation on the urinary apparatus	$1,167,588	$303,286	$1,470,874	$1,438,278	$541,380	$1,979,659
Operation on the female genital apparatus	$1,599,337	$ -	$1,599,337	$1,971,837	$ -	$1,971,837
Procedures performed on the central nervous system	$1,630,076	$514,165	$2,144,242	$1,360,441	$434,293	$1,794,735
Procedures performed on the endocrine system	$703,628	$76,204	$779,832	$377,999	$ -	$377,999
Operation on the hematic and/or lymphatic system	$184,876	$105,308	$290,184	$296,615	$ -	$296,615
Ear operation and related procedures	$141,625	$77,279	$218,904	$218,904	$77,278	$296,182
Operation on the male genital apparatus	$ -	$142,183	$142,183	$ -	$184,841	$184,841
Obstetric procedures	$66,229	$ -	$66,229	$66,229	$ -	$66,229
Total	$144,518,863	$25,580,927	$170,099,794	$136,486,772	$30,552,702	$167,039,481

^++^ The IMSS category is “diagnostic tests and miscellaneous”, a misleading title. Exchange rate (1 USD = 18.7 MXN)

## Discussion

RA is a chronic disease that involves a high level of clinical attention, taking into account the average of the attention given to patients. Consequently, this disease consumes a large amount of hospital resources. During the evolution of the disease, patients pass through different stages of drug therapy (synthetic and biological). Among RA patients, a relatively high percentage require medical procedures and surgical interventions. The present study analyzed the total IMSS hospital resources dedicated to inpatient care of beneficiaries with RA, finding a total cost of $170,099,794 MXN ($9,096,245.67 USD) for 2016 and $167,039,481 MXN for ($8,932,592.57 USD) 2017.

Since the hospital expenses for RA are known to be substantial, it is necessary to determine the real cost of the disease for each institution. However, there is scant data in the literature on the economic impact of RA for public hospitals. Kannayiram et al. and Gil-Conesa et al. described the cost of drug therapy for RA by utilizing ICD-9-CM and ICD-10 [22, 23], but did not investigate the expenditure of hospital resources for medical procedures and surgical interventions.

Lajas et al. examined the costs involved in treating RA in a cohort of 342 patients in the Hospital Clínico San Carlos in Madrid, Spain. The author classified the patients with the CIE-10 system and assessed direct and indirect costs, finding that medical costs account for about 60% of the economic burden of RA for the hospital. The average total annual cost per patient was ~ 11,300 USD [24].

Hernández-Cruz et al. (2006) focused on a cohort of 90 patients with RA in the Instituto Nacional de Ciencias Médicas y Nutrición “Salvador Zubirán” in Mexico City, Mexico, evaluating the direct costs of standard treatment in outpatient care. Such costs consisted of medical consultation, medications, and lab and diagnostic tests. The cost of drug treatment was calculated based on price tables of the institution for 1998. The cost of the standard treatment of RA was established as $152,704.11 MXN annually per patient adjusted to 2005 prices ($1,735.27 USD, considering the relevant exchange rate), of which 51% corresponded to medical attention, 25.76% to drug treatment, and 22.91% to other direct costs [25]. In 1998, only synthetic drug therapy existed for treating RA, as biological medications had not yet been developed. Hence, the cost of therapy was different then than today.

The present results show an approximate total cost of $132,053,692 MXN in 2016 and $130,144,272 MXN in 2017 to treat all inpatient RA-related procedure in those years, indicating a slight decline in overall costs compared to the previously cited study. Nevertheless, the $1,909,420 MXN reduction is a marginal and economically irrelevant difference of ~ 2%. Considering the American College of Rheumatology Guideline (ACR 2021) as a reference and assuming that the patients take an average of three boDMARDs (the most common in the public sector in Mexico being Adalimumab, Etanercept, and Tocilizumab), the range of costs annually per patient would be from $14,500 to $50,400 MXN [26].

Álvarez-Hernández et al. analyzed the expenses for the families of 262 patients with RA in Mexico, finding the impoverishment of the respective households. The average annual cost of the medical attention was $3,599 ± 6,621 USD, while the indirect costs were $1,409 ± 6,621USD (using 2005 prices updated to 2012). Regarding the economic impact of RA for the family of each patient, an average of 65% of family income was destined to out of pocket expenses for the purchase of products related to RA [27]. The focus on the expenses of the family of RA patients is outside the scope of the current contribution.

Among the different techniques for the assessment of costs [28], the DRG method was adopted presently. It was recommended by the Professional Society for Health Economics and Outcomes Research (formerly the International Society for Pharmacoeconomics and Outcomes Research, or ISPOR) in their consolidated report on standards for the description of economic evaluations in healthcare (CHEERS 2022). The method is based on a generalized approximation of costing without the need for detailing the specific resources utilized [29]. Although another method, micro-costing, reduces the uncertainty in the results, the nature of the data currently available did not allow for its use.

The limitation of this study is the method of costing, which only provides an approximation and therefore affects the accuracy of the institutional costs to some degree. Since the methodology assumes that the patients do not have important comorbidities with RA, the results may reflect an underestimation of the real total costs per patient. However, the methodology was in accordance with the present aim of making an estimate of the cost of RA isolated from comorbidities. Another limitation was the reliance on databases, which are created through a digital transcription of the original physical copy of the data. Even though the transcription is carried out by specialists with systematic techniques, the chance of error implies a potential bias inherent in the methodology.

It is important to note that our study primarily focuses on direct costs within the institutional perspective of IMSS. We understand that there are additional economic factors associated with RA, including indirect costs such as those related to lost productivity and the financial impact on patients and their families. While our research contributes valuable insights into the intrahospital phase of RA care, we acknowledge that this study does not encompass the broader economic consequences of RA comprehensively. We recognize the need for future research to explore the full spectrum of economic implications, including both direct and indirect costs. This limitation highlights the complexity of capturing the complete economic burden of RA and underscores the importance of further investigations in this area.

In relation to the strengths of this investigation, the sample population was representative of the overall Mexican population and the database belonged to the largest healthcare system in the country. Additionally, it was herein demonstrated that diseases like RA can produce a great economic impact on the government budget for the health sector. An underestimation of the real economic impact associated with RA negatively affects an optimal government allotment for the disease and consequently affects the quality of care for the corresponding patients. That being the case, the determination of the real overall costs associated with a particular disease is now an essential consideration of the UNICEF when planning its annual health budget [30].

In future research, a more detailed economic study should be conducted by utilizing micro-costing techniques in order to attain a more accurate appraisal of the costs of inpatient care and drug treatment (both synthetic and biologic) for persons afflicted with RA. It would then be possible to make a definitive finding about whether or not RA is a disease with catastrophic expenses for the Mexican healthcare system.

## Conclusion

The medical procedures responsible for the greatest consumption of hospital resources by patients with RA in the IMSS during 2016–2017 were instrumented procedure on the musculoskeletal apparatus and cardiovascular surgery. The cost of inpatient care for adults with RA was presently found to produce an enormous economic impact on the budget of the Mexican healthcare system. Further research is needed on the cost of drug treatment and the indirect costs associated with RA to ascertain a more precise assessment of the economic burden of this disease, which very probably involves catastrophic expenses for the Mexican healthcare system. This study could provide strong data to support budget allocation to treat RA patients.

### Electronic supplementary material

Below is the link to the electronic supplementary material.

Supplementary Material 1

## Data Availability

The datasets analysed during the current study are available in the Información en Salud | Datos Abiertos IMSS repository, http://datos.imss.gob.mx/dataset/informaci%C3%B3n-en-salud. Full access is restricted to intranet users, for further details the datasets are available from the corresponding author on reasonable request.

## List of Abbreviations

DRG: Diagnosis-related group
RA: Rheumatoid arthritis
DALYs: Disability-adjusted life years
csDMARDs: conventional synthetic disease-modifying antirheumatic drugs
boDMARDs: biological disease-modifying antirheumatic drugs
IRT: inadequate responses to treatment
IMSS: Instituto Mexicano del Seguro Social
