# Supplementary material for: Health and economic impact associated with rheumatoid arthritis discharges: a cost analysis of a two-year cohort in Mexico
Source: BMC Health Serv Res. 2023 Nov 29;23:1320. doi: 10.1186/s12913-023-10298-w (PMC10687957; doi:10.1186/s12913-023-10298-w)
Supplement: Supplementary file 1 — Supplementary Material 1 [file 12913_2023_10298_MOESM1_ESM.pdf]

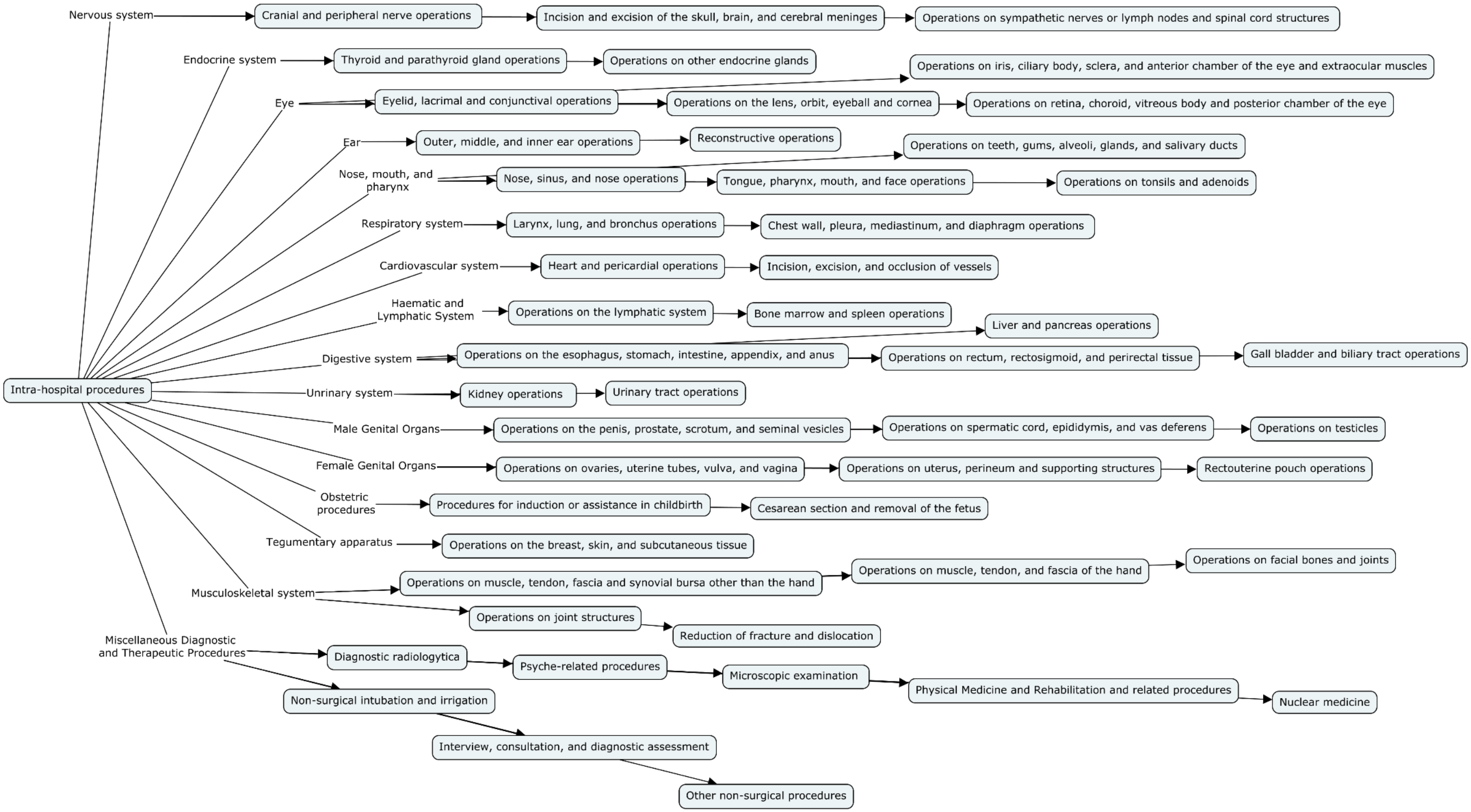

Supplementary figure 1. Intra-hospital procedures. A brief summary of the 16 medical procedures performed on each patient according to the ICD-9-CM Classification of Procedures (published by the Mexican Ministry of Health).
